# Exposure to Bisphenol B and S Increases the Risk of Male Reproductive Dysfunction in Middle Age

**DOI:** 10.3390/ijms26199507

**Published:** 2025-09-28

**Authors:** Sen Zhao, Heliang Ni, Yuan Xiao, Jing Du, Yudong Han, Wenying Wang, Shuang Tang, Mingxi Yu

**Affiliations:** 1 College of Bioscience and Biotechnology, Shenyang Agricultural University, Shenyang 110866, China; senzhao200103@gmail.com (S.Z.);; 2 Key Laboratory of Protection and Utilization of Aquatic Germplasm Resources, Ministry of Agriculture and Rural Affairs, Liaoning Ocean and Fisheries Science Research Institute, Dalian 116023, China

**Keywords:** network toxicology, bisphenol B, bisphenol S, middle-age, male reproduction dysfunction

## Abstract

Accumulating evidence indicates that bisphenol A (BPA) analogs, including bisphenol B (BPB) and bisphenol S (BPS), disrupt testicular function and contribute to male reproductive dysfunction (MRD). However, whether BPA analogs are involved in MRD among middle-aged men remains inconclusive. Therefore, we selected cryptorchidism, erectile dysfunction, premature ejaculation, and testicular tumors as representative MRD conditions in middle-aged individuals, aiming to explore the molecular mechanisms that may be disrupted by bisphenols (BPs). By using GeneCards, STRING and Cytoscape, TP53, AKT1, and MYC were pinpointed as core targets associated with MRD. Enrichment analysis suggested that BPs may induce MRD by disrupting steroidogenesis. UPLC-MS/MS analysis showed that both BPB and BPS exhibit specific accumulation in the testes. Following 20-day exposure to 0.3 or 0.6 mg/kg body weight/day BPB or BPS, testosterone levels and the expression of hub genes were decreased. The molecular docking results demonstrated that both BPB and BPS can directly bind to members of the cytochrome P450 family, potentially interfering with sex hormone biosynthesis. Our study identified the targets and mechanisms through which BPB and BPS induce MRD in middle-aged males, thereby providing insights for the safety assessment of BPs.

## 1. Introduction

Bisphenol A (BPA) is one of the most extensively used plasticizers in the production of epoxy resins and polycarbonate resins [1,2]. A growing number of studies indicated that BPA could be found in urine, maternal amniotic fluid, and serum [3,4,5,6]. Nevertheless, accumulating evidence has identified that BPA can lead to a range of diseases, including reproductive disorders, immune dysfunction, neurobehavioral impairments, carcinogenic hypersensitivity reactions, etc. As a result, BPA has been replaced with its analogs compounds [7,8], notably bisphenol B (BPB) and bisphenol S (BPS). Numerous studies have revealed that BPB and BPS are widely present in environmental and biological samples, with growing evidence showing their levels exceed those of BPA in both the environment and ecosystem [9]. BPB and BPS exert endocrine-disrupting effects, including estrogenic and anti-androgenic activities [10,11]. Specifically, BPB has been demonstrated to significantly reduce serum testosterone levels while upregulating gonadotropins, indicating disruption of the hypothalamic–pituitary–gonadal (HPG) axis and subsequent alterations in reproductive development [12,13]. Furthermore, BPB downregulates androgen receptor (AR) expression and genes involved in spermatogenesis, potentially via estrogen receptors (ER) ER-α or ER-β activation, thereby antagonizing androgen signaling pathways [14]. Additionally, BPS interferes with reproductive hormone homeostasis by directly inhibiting the lyase activity of cytochrome P450 family 2 subfamily C member 9 (CYP2C9), which impairs the conversion of androgens to estrogens in human adrenocortical carcinoma cell [15].

The reproductive system is one of the key targets of bisphenol (BP) exposure [16,17,18]. BPB and BPS can disrupt normal physiological structures and functions through multiple mechanisms, ultimately contributing to male reproductive dysfunction (MRD). MRD, a multifactorial disorder commonly seen in middle-aged men, is generally classified into three major categories: 1. Sperm formation-related pathologies (e.g., oligoasthenoteratozoospermia, azoospermia); 2. Sexual function disorders (e.g., erectile dysfunction); 3. Organic diseases (e.g., undescended testes) [19,20]. The molecular mechanisms underlying MRD include oxidative stress, hormonal dysregulation, genetic and epigenetic alterations, immune imbalance, and disruptions in cell death pathways. Cryptorchidism is one of the most prevalent congenital anomalies of the male genital system, associated with factors such as low birth weight, prematurity, and maternal smoking during pregnancy. Additionally, endocrine disrupting chemicals (EDCs) may disrupt the normal process of testicular descent [21,22]. Erectile dysfunction (ED) is a clinical condition that predominantly affects men over 40 years of age, often associated with underlying factors such as diabetes mellitus, hypertension, insufficient physical activity, and lower urinary tract symptoms [23,24]. Premature ejaculation (PE) is most regulated by both the central and peripheral nervous systems [25,26]. This condition not only causes psychological distress, but also significantly impairs male fertility, with clinical manifestations, including anejaculation, reduced ejaculatory volume, retrograde ejaculation, painful ejaculation, or premature ejaculation [27]. Testicular tumors (TT) represent the most prevalent neoplasm diagnosed in men aged 15 to 44 years, originating from germ cell neoplasia in situ. In utero exposure to EDCs has been implicated as a potential factor influencing offspring susceptibility to testicular tumors later in life, highlighting the complex interaction between environmental exposures and disease pathogenesis [28,29]. These conditions are influenced by a range of genetic, hormonal, neural, psychological, and environmental factors that disrupt spermatogenesis, hormonal homeostasis, and sexual function, thereby exerting significant detrimental effects on male fertility and reproductive health. Despite growing evidence of the reproductive toxicity of BPB and BPS, their specific impacts on middle-aged males remain poorly characterized—highlighting a critical knowledge gap that warrants further investigation.

Network toxicology has emerged as an interdisciplinary methodology that integrates bioinformatics, genomics, multi-omics technologies, and data analytics to systematically elucidate the complex interactions among compounds, molecular targets, and biological pathways. It offers a holistic framework to unravel intricate toxicological mechanisms [30]. It provides a holistic framework for unraveling intricate toxicological mechanisms. By conducting in silico analyses of human genes, we identified core potential molecular mechanisms, which were further investigated using mouse disease models. This integrated strategy thereby enables accurate and cost-effective exploration of the underlying mechanisms. In the present study, we employed network toxicology, molecular docking techniques, steroid hormone synthesis assays, histopathological analyses, and expression profiling of steroidogenesis-related genes. The objective of this study was to employ network toxicology and molecular docking methodologies to elucidate the molecular mechanisms underlying the effects of BPB and BPS on MRD. Furthermore, five-month-old male mice were utilized as an experimental model representative of middle-aged men, providing empirical data on the toxicological impact of these environmental contaminants.

## 2. Result

### 2.1. Landscape of Genes Co-Expressed in BPB and BPS

**Figure 1:**
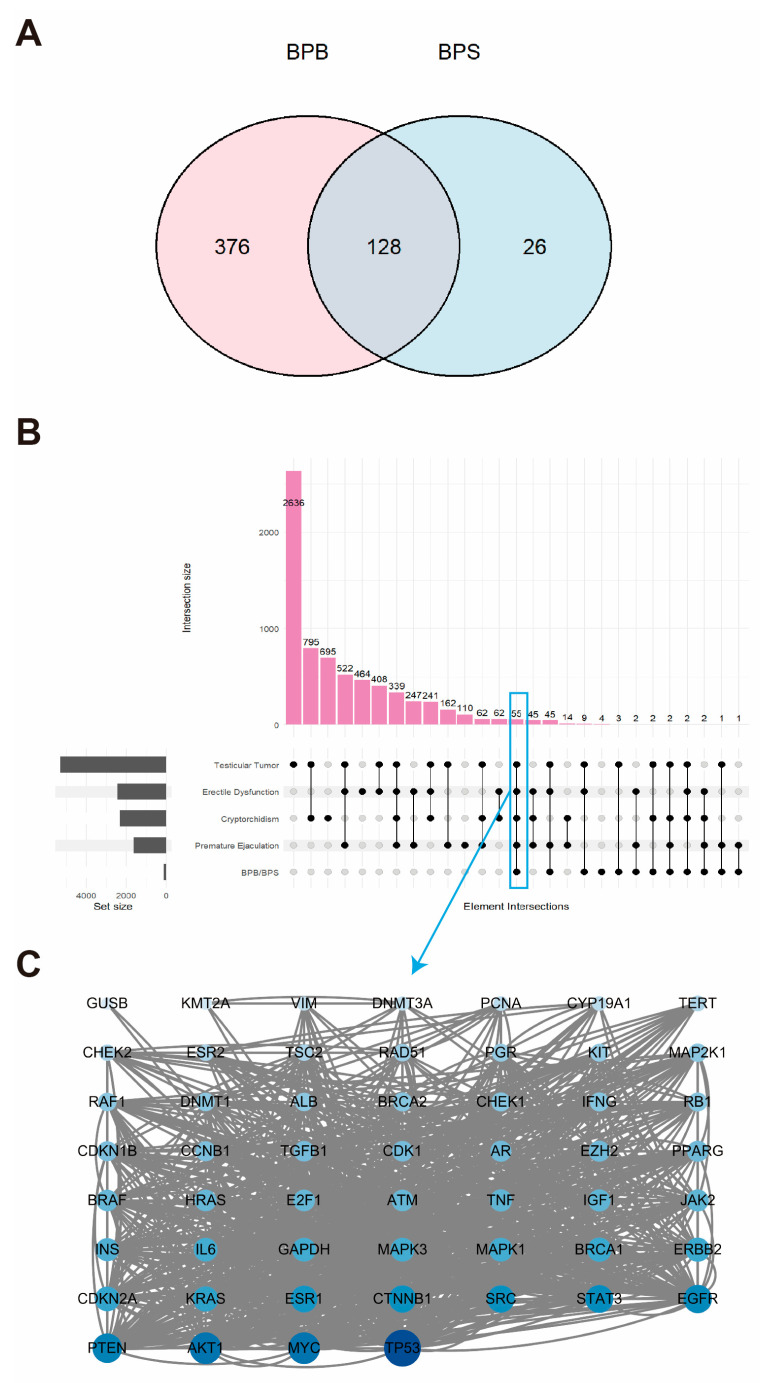
Genes co-expressed in BPB and BPS. (**A**) Venn diagram illustrating the overlapping target genes of BPB and BPS identified from the GeneCards database. (**B**) Upset plot showing the intersection of gene targets among four MRDs. The pink bars represent the quantity of genes within each set. The black dots denote the associated MRD disease sets. And the gray histogram illustrates the gene counts across the four MRD diseases. (**C**) PPI network of potential therapeutic targets associated with MRDs, constructed using the STRING database and visualized with Cytoscape. Each node represents a protein. The size and color intensity of the nodes indicate the number of interactions, with larger and darker nodes representing proteins that interact with more partners.

A comprehensive screening program was conducted to identify 504 BPB-related targets and 154 BPS-related targets in humans from the GeneCards database. These targets were integrated to generate a Venn diagram and a BPs–target network, which illustrate the overlap between BPB and BPS targets (Figure 1A, Table S2). A total of 128 overlapping targets were identified for BPB and BPS. Additionally, potential MRD-related targets in humans were retrieved from the GeneCards database. Disease-specific targets associated with four representative MRD conditions—cryptorchidism, ED, TT, and PE—were compiled, and an UpSet plot was constructed to visualize their overlap. A total of 55 common targets were identified across these four conditions (Figure 1B). Further analysis revealed three hub proteins—AKT1, MYC, and TP53—as key targets associated with MRD in humans (Figure 1C).

### 2.2. Network Toxicology Analysis of Potential Target Genes Commons to Both Cryptorchidism and BPs

**Figure 2:**
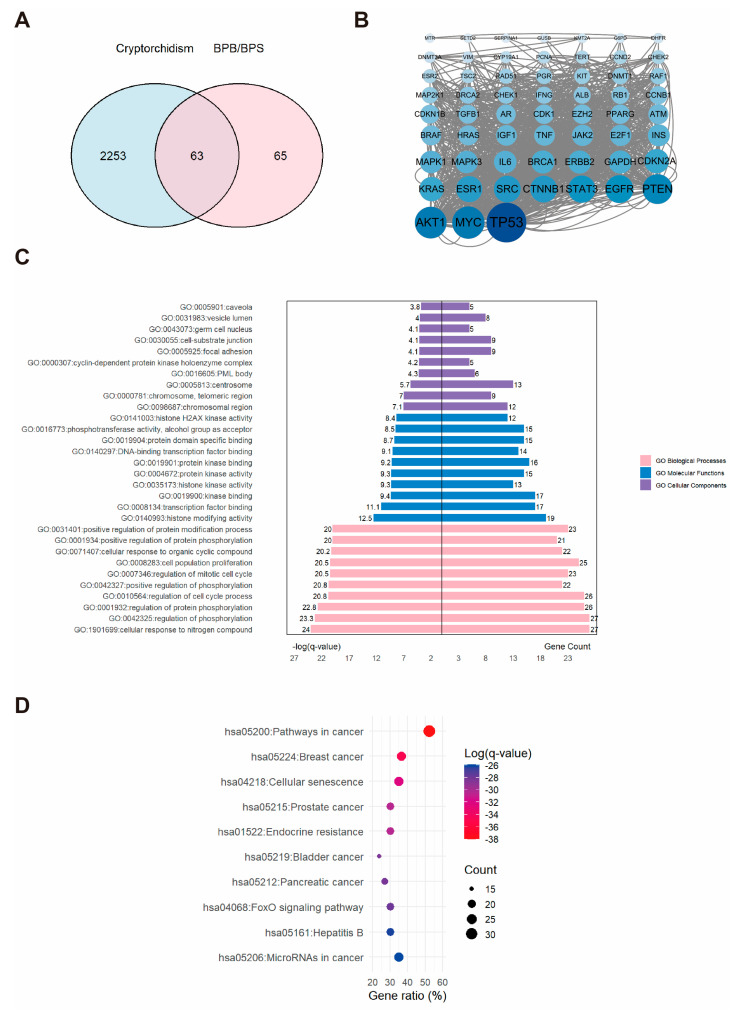
The associations between BPs and cryptorchidism. (**A**) Venn diagram showing the overlapping targets between BPs exposure and cryptorchidism-associated genes. (**B**) PPI network constructed from the potential therapeutic targets of BPs in cryptorchidism. Each node represents a protein. The size and color intensity of the nodes indicate the number of interactions, with larger and darker nodes representing proteins that interact with more partners. (**C**) GO enrichment analysis and (**D**) KEGG pathways enrichment analysis of the core targets. “GO numbers” indicate Gene Ontology term identifiers, and “Hsa” denotes *Homo sapiens* pathways.

A total of 2316 potential cryptorchidism-related genes were identified, exhibiting diverse biological functions. A Venn diagram revealed the overlap between cryptorchidism-related genes and BPs targets (Figure 2A), highlighting 63 common targets specifically linked to BPs-induced cryptorchidism. A PPI network was constructed for these 63 potential targets and subsequently analyzed with Cytoscape (Figure 2B). And three proteins, AKT1, MYC, and TP53, were identified as the core genes. KEGG analysis indicated that these genes were involved in 157 pathways. GO analysis identified 1251 terms in Biological Process (BP), 65 in Cellular Component (CC), and 150 in Molecular Function (MF). The top ten enriched terms in each category were visualized using a butterfly bar plot. As illustrated in Figure 2C, the BP category was predominantly associated with cellular response to nitrogen compound. The CC category was chiefly related to the “chromosomal region”. The MF category was largely concerned with “histone modifying activity”. Figure 2D demonstrated that the most significantly enriched KEGG pathway was the “pathways in cancer”. These findings provide valuable insights into the potential molecular mechanisms underlying BPs-induced cryptorchidism.

### 2.3. Network Toxicology Analysis of Potential Target Genes Commons to Both Erectile Dysfunction and BPs

**Figure 3:**
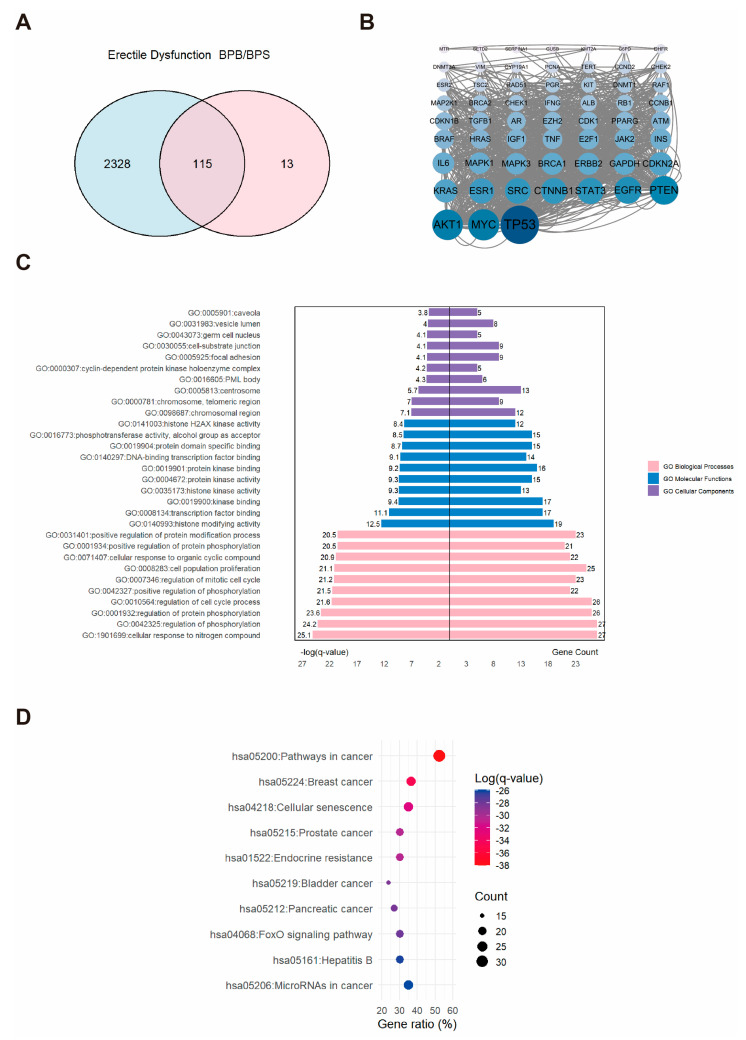
The associations between BPs and ED. (**A**) Venn diagram showing the overlapping targets between BPs exposure and ED-associated genes. (**B**) PPI network constructed from the potential therapeutic targets of BPs in ED. Each node represents a protein. The size and color intensity of the nodes indicate the number of interactions, with larger and darker nodes representing proteins that interact with more partners. (**C**) GO enrichment and (**D**) KEGG pathways enrichment analysis of the core targets. “GO numbers” indicate Gene Ontology term identifiers, and “Hsa” denotes *Homo sapiens* pathways.

A comprehensive dataset comprising 2443 ED-related targets was obtained and subsequently integrated with the 128 BP-associated targets (Figure 3A). This analysis delineated the intersection between BPs and ED targets, identifying 115 overlapping targets potentially involved in BPs-induced erectile dysfunction. To further investigate these targets, a PPI network was constructed and visualized in Cytoscape software. We identified 115 core targets, and the top three hub proteins identified were AKT1, MYC, and TP53 (Figure 3B). Subsequent functional enrichment analysis yielded a substantial dataset, comprising 156 pathways and 2222 GO terms, which encompassed 2007 BP, 65 CC, and 150 MF. The top ten GO terms in each category were selected based on the highest −log(*q*-value) and visualized accordingly (Figure 3C). Within the BP category, the predominant association was with the positive regulation of the “cellular response to nitrogen compound”. The CC category was chiefly linked to the “chromosomal region”. The MF category was primarily related to “histone modifying activity”. For KEGG analysis, the top ten enriched pathways were selected and displayed using an enrichment bubble plot, providing insights into the mechanisms underlying erectile dysfunction and BPs (Figure 3D).

### 2.4. Network Toxicology Analysis of Potential Target Genes Commons to Both Premature Ejaculation and BPs

**Figure 4:**
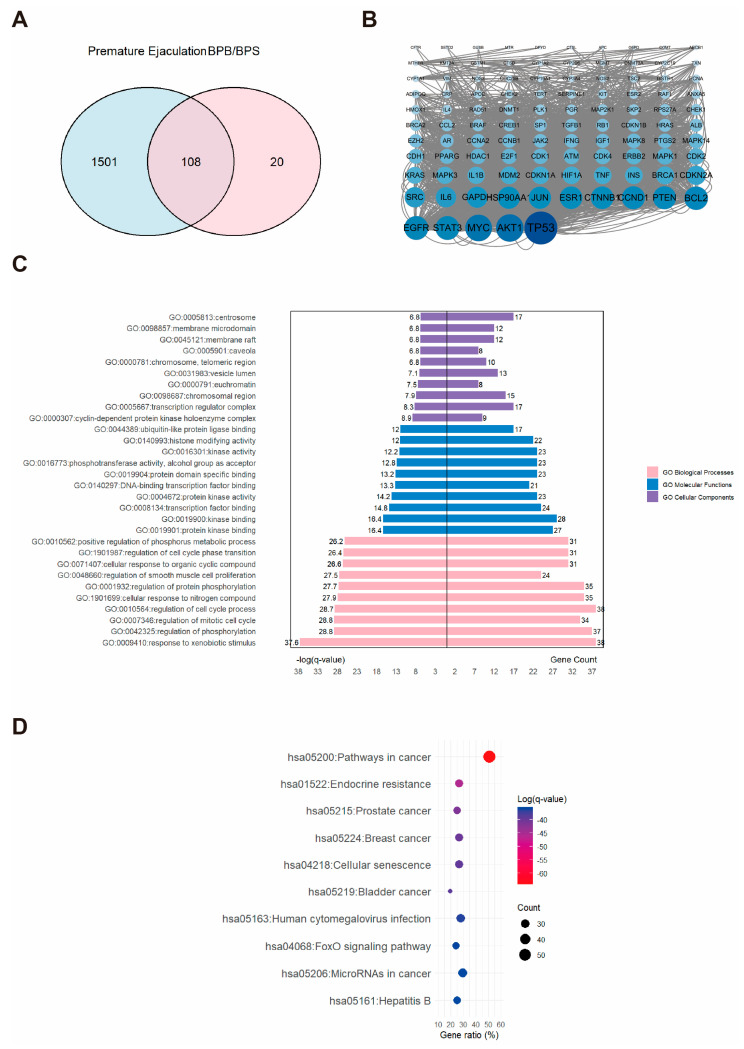
The associations between BPs and PE. (**A**) Venn diagram showing the overlapping targets between BPs exposure and PE-associated genes. (**B**) PPI network constructed from the potential therapeutic targets of BPs in PE. Each node represents a protein. The size and color intensity of the nodes indicate the number of interactions, with larger and darker nodes representing proteins that interact with more partners. (**C**) GO enrichment and (**D**) KEGG pathway enrichment analysis of the core targets. “GO numbers” indicate Gene Ontology term identifiers, and “Hsa” denotes *Homo sapiens* pathways.

A total of 1609 PE-related targets were identified and subsequently integrated with BP-associated targets (Figure 4A). Revealing an intersection of 108 overlapping targets that were considered potential candidates for BPs-induced PE. PPI network was constructed, and the top three hub proteins were identified as AKT1, MYC, and TP53 (Figure 4B). These 108 core targets were further enriched through GO and KEGG analysis, which revealed their involvement in 1737 BP, 100 CC, and 183 MF GO terms, as well as 184 KEGG pathways. As shown in Figure 4C, a butterfly bar plot was employed to visualize the top ten enriched terms in each GO category, ranked by −log(*q*-value). The BP category was primarily associated with “response to xenobiotic stimulus”. The CC category was mainly related to the “cyclin-dependent protein kinase holoenzyme complex”. The MF category was largely concerned with “kinase binding”. The top three enriched KEGG pathways were identified as “pathways in cancer,” “endocrine resistance,” and “prostate cancer” (Figure 4D).

### 2.5. Network Toxicology Analysis of Potential Target Genes Commons to Both Testicular Tumor and BPs

**Figure 5:**
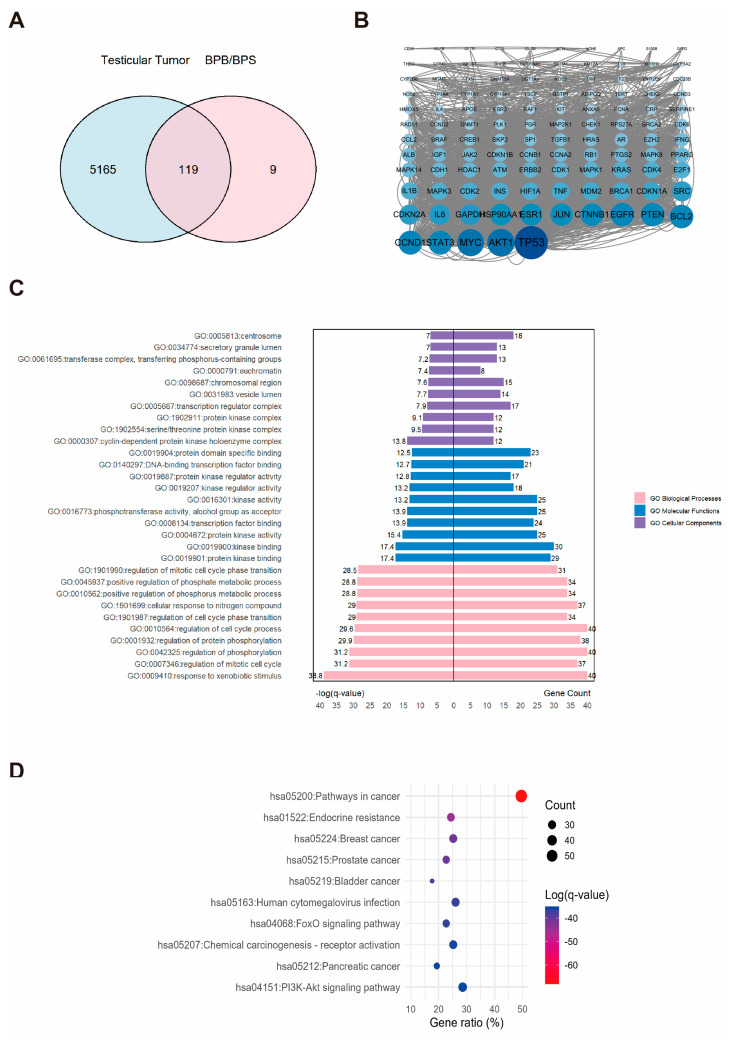
The associations between BPs and TT. (**A**) Venn diagram showing the overlapping targets between BPs exposure and TT-associated genes. (**B**) PPI network constructed from the potential therapeutic targets of BPs in TT. Each node represents a protein. The size and color intensity of the nodes indicate the number of interactions, with larger and darker nodes representing proteins that interact with more partners. (**C**) GO enrichment and (**D**) KEGG pathways enrichment analysis of the core targets. “GO numbers” indicate Gene Ontology term identifiers, and “Hsa” denotes *Homo sapiens* pathways.

A total of 5284 TT-related targets were identified and subsequently integrated with the BP-associated targets (Figure 5A). This diagram revealed an intersection comprising 119 overlapping targets, which were considered potential candidates associated with BPs–induced TT (Figure 5A). A PPI network was constructed for these 119 potential targets by using the STRING database and subsequent analysis with Cytoscape (Figure 5B). We identified the AKT1, MYC, and TP53 as the hub proteins (Figure 5B). Functional enrichment analysis was performed. and generated a dataset, including 190 pathways and 2033 GO entries, which encompassed 1746 BP, 101 CC, and 186 MF. A butterfly bar plot was employed to visualize the top ten enriched terms in each GO category, ranked by −log(*q*-value). As shown in Figure 5C, the BP category was primarily associated with “response to xenobiotic stimulus”. The CC category was mainly related to the “cyclin-dependent protein kinase holoenzyme complex”. The MF category was largely concerned with “kinase binding”. The top one enriched KEGG pathways were identified as “pathways in cancer” as the most significantly enriched pathway (Figure 5D).

### 2.6. Molecular Docking of BPs with Potential Target Proteins

**Figure 6:**
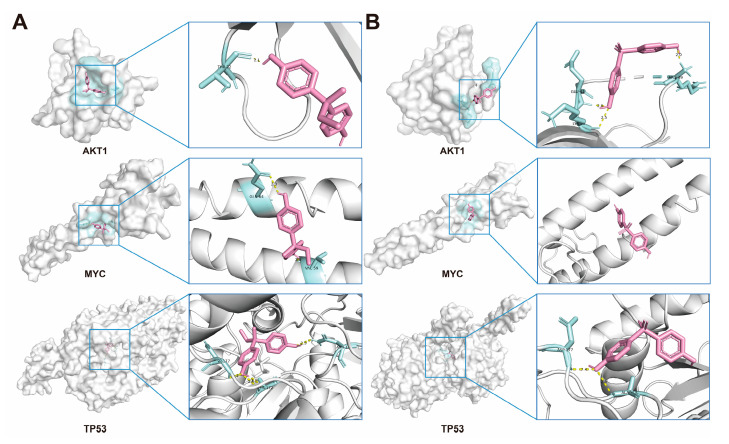
Molecular docking of BPs with potential receptors. (**A**) Docking results of AKT1, MYC and TP53, with the BPB, respectively. (**B**) Docking results of AKT1, MYC and TP53, with the BPS, respectively. BPB or BPS molecules are depicted in red.

Based on the results of PPI screening, AKT1, MYC, and TP53 were selected to assess the interactions between BPs and these targets. The most probable binding conformations showed that BPB could bind tightly to AKT1, MYC, and TP53, with binding energies of −6.0, −5.9 and −7.3 kcal/mol, respectively, indicating a high level of binding stability. Further analysis revealed that the binding was characterized by at least two hydrogen bonds formed with the residues of each target, as well as hydrophobic interactions and potential electrostatic forces, demonstrating strong interaction forces. Similar results were observed following BPS exposure, with the exception of MYC. These findings indicate that MRD exhibits broad binding affinity for both BPB and BPS—likely attributable to its functional role in transporting diverse small molecules within the human body (Figure 6, Table 1). Notably, with the exception of AKT1 and BPS-related regulation—which are more likely to be indirectly modulated rather than directly bound—all other core targets demonstrate direct robust molecular docking interactions. This suggests that the tight binding of BPB and BPS to these receptors may play a critical role in mediating the molecular mechanisms underlying MRD.

### 2.7. BPs Concentration in Serum and Testis

**Figure 7:**
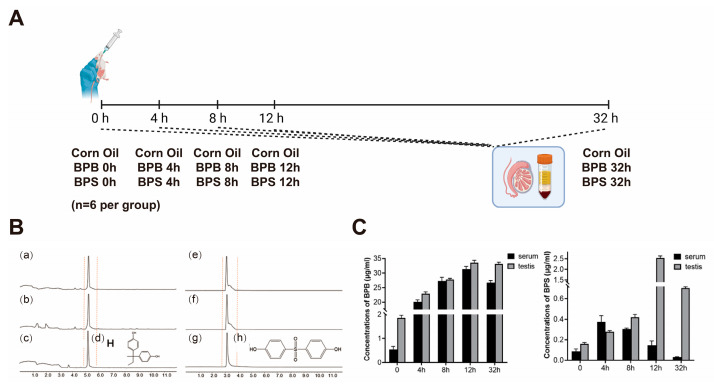
Qualitative and quantitative analyses of BPs in mouse serum and testis. (**A**) For the single gavage administration, male mice were randomly divided into fifteen groups (*n* = 6 per group): a corn oil group (negative control) and BPB- and BPS-treated groups. Experimental sampling was performed at 0, 4, 8, 12, and 32 h post-administration. (**B**) UPLC-MS/MS analysis of BPB and BPS in mouse serum and testis, including representative chromatograms and chemical structures of BPB and BPS (100 μg/mL). (**a**–**c**) UPLC-MS/MS analysis of BPB in mice blood, mice testis, and BPB. (**d**) Chemical structures of BPB. (**e**–**g**) UPLC-MS/MS analysis of BPS in mice blood, mice testis, and BPS. (**h**) Chemical structures of BPS. (**C**) Concentrations of BPB and BPS in serum and testis following exposure. Data are presented as mean ± SEM (*n* = 3 per group).

The concentrations of BPB and BPS in serum and testicular tissues were quantified via UPLC-MS/MS analysis (Figure 7A,B). Following a single high-dose gavage administration, both BPB and BPS were detected in the serum and testes of mice, confirming their distribution in reproductive organs and revealing limited metabolic clearance within 32 h (Figure 7C). Moreover, during the exposure period, BPB and BPS were eliminated from the serum more rapidly than from the testes, indicating tissue-specific retention properties.

### 2.8. Serum Hormone Levels and Morphological Changes in the Testis in Middle-Aged Male Mice

**Figure 8:**
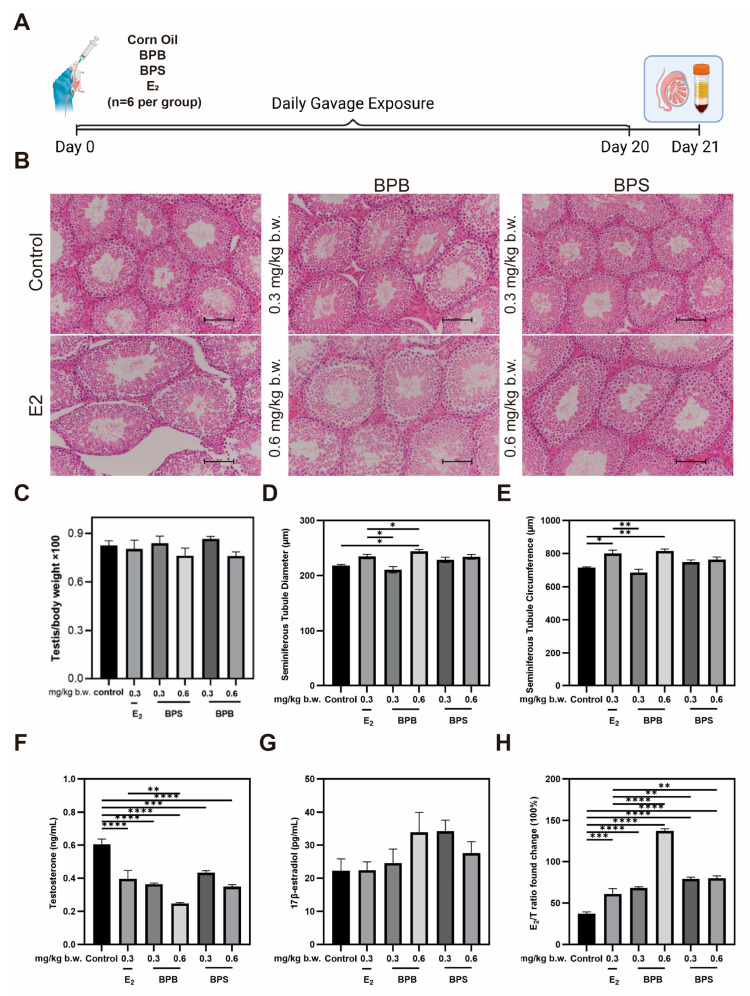
Changes in the testicular morphology and sex hormone levels of BPB- and BPS-exposed mice. (**A**) For the sub-chronic gavage administration, male mice were randomly divided into six groups (*n* = 6 per group): 0 mg/kg body weight (b.w.) (control), 0.3 mg/kg b.w. BPB, 0.6 mg/kg b.w. BPB, 0.3 mg/kg b.w. BPS, 0.6 mg/kg b.w. BPS, and 0.3 mg/kg b.w.17β-estradiol (E_2_). (**B**) Representative images of H&E staining (Scale bar = 100 μm) in mice testis sections after 20-day exposure. (**C**) Ratio of testis and body weight (testis/body weight × 100%) were examined. (*n* = 6). (**D**) Quantitative analysis of seminiferous tubule diameter and (**E**) seminiferous tubule circumference of the testis in mice (*n* = 6). (**F**) T and (**G**) E_2_ level with BPB or BPS. (**H**) Ratio of E_2_ and T (17β-estradiol/testosterone weight × 100%) were examined. The values are recorded as the means ± SEM. * p < 0.05, ** p < 0.01, *** p < 0.001 and **** p < 0.0001 compared with the corresponding control group.

The effects of BPB and BPS on body weight and relative testis weight were examined (Figure 8C), and neither BPB nor BPS had a significant effect on mouse body weight. Compared with the control group, relative testis weight decreased following exposure to 0.6 mg/kg b.w. BPB or 0.6 mg/kg b.w. BPS, though these differences were not statistical significance. Histopathological examination revealed that the testes in the control group displayed tightly arranged seminiferous tubules with thickened epithelial layers, lumens filled with spermatozoa, and thin, round interstitial spaces. In contrast, estradiol-treated mice exhibited reduced spermatozoa in the lumens, enlarged luminal cavities, and oval-shaped seminiferous tubules (Figure 8B). Similarly, in mice treated with BPB and BPS at 0.3 mg/kg and 0.6 mg/kg body weight, a reduction in intraluminal spermatozoa and a progressive shift toward oval-shaped tubules were also observed. These findings indicate that exposure to BPB and BPS in middle-aged mice, in a dose-dependent manner. BPB and BPS likely could impair spermatogonial development and spermatogenic differentiation, ultimately reducing sperm count and altering testicular architecture. The effect of BPB and BPS on seminiferous tubule diameter was significant, with a 7.2% and 11.8% increase observed in the 0.6 mg/kg/day BPB and 0.6 mg/kg/day BPS exposure groups (Figure 8D), respectively. The seminiferous tubule circumference increased following exposure to 0.6 mg/kg/day BPB, which was 14.2% higher than that in the control group (Figure 8E). These results suggest that BPB and BPS may induce testicular damage. Serum hormone levels showed that, compared with the control group, BPB and BPS significantly decreased the level of testosterone (T) in serum after 20-day exposure (Figure 8F). However, 17β-estradiol (E_2_) levels increased to varying degrees in treatment groups (Figure 8G). The E_2_/T ratios were significantly higher in all treatment groups relative to the control, indicating disrupted endocrine function in middle-aged male mice (Figure 8H).

### 2.9. Identification of Potential Targets of Steroidogenic Enzyme Expression

**Figure 9:**
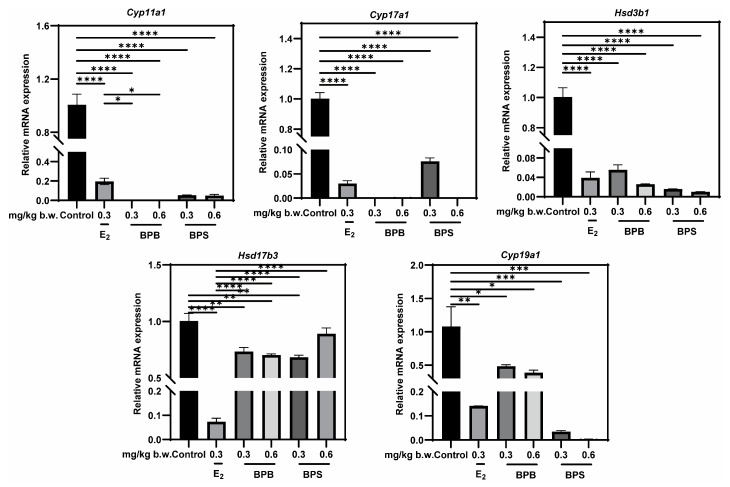
Effects of exposure to BPB or BPS on the expression of steroidogenic enzyme in middle-age male mice. The mRNA levels of *Cyp11a1*, *Cyp17a1*, *Hsd3b1*, *Hsd17b3* and *Cyp19a1* were measured in testicular tissue using RT-qPCR. *Gapdh* served as an internal control. The values are recorded as the means ± SEM. * *p* < 0.05, ** *p* < 0.01, *** *p* < 0.001 and **** *p* < 0.0001 compared with the corresponding control group.

Alterations in sex hormone levels may be associated with changes in the expression of genes involved in steroid biosynthesis [31,32]. The RT-qPCR results revealed a significant downregulation in the expression of these five key steroidogenic enzyme genes following exposure to BPB or BPS, including *Cyp11a1*, *Cyp17a1*, *Cyp19a1*, *Hsd3b1*, and *Hsd17b3* (Figure 9). These findings, consistent with the network toxicology analysis, underscore the critical role of hormones in BPs-induced male reproductive damage (MRD).

### 2.10. Verification of Interactions Between BPs and Potential Targets Through Molecular Docking

**Figure 10:**
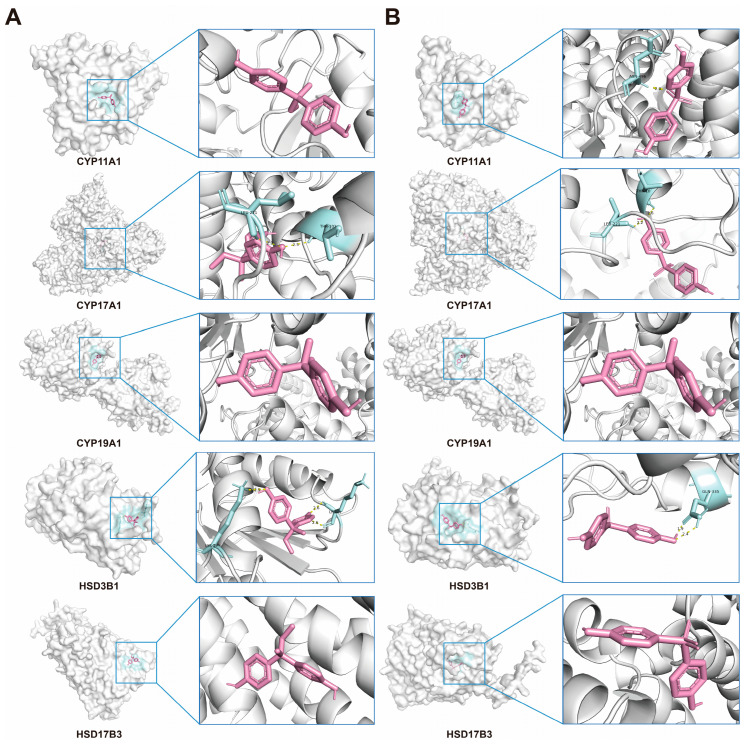
Molecular docking of BPs with Human steroidogenic enzyme. (**A**) Docking results of CYP11A1, CYP17A1, CYP19A1, HSD3B1, and HSD17B3, with the BPB, respectively. (**B**) Docking results of CYP11A1, CYP17A1, CYP19A1, HSD3B1, and HSD17B3, with the BPS, respectively. BPB or BPS molecules are depicted in red.

Molecular docking analyses were conducted to evaluate the interactions between BPB, BPS and five key steroidogenic enzymes: CYP11A1, CYP17A1, CYP19A1, HSD3B1, and HSD17B3. Both BPB and BPS exhibited strong binding affinities toward CYP17A1, CYP19A1 and HSD17B3, while BPB also demonstrated high-affinity binding to CYP11A1 and HSD3B1, as indicated by docking scores below −5.0 kcal/mol. These results indicated that bisphenols possess the strong binding capacity for critical proteins involved in steroidogenesis, underscoring their potential role in mediating endocrine disruption. To further characterize the binding conformations, PyMOL was employed to visualize the lowest-energy docking poses (Figure 10, Table 2). These structures reveal close-fitting interactions between bisphenols and their respective protein targets.

## 3. Discussion

The widespread application of bisphenols has raised concerns regarding their accumulation in biological systems and the increased incidence of adverse reproductive outcomes in males [1,33]. BPs has been documented to disrupt testicular steroidogenesis, with steroidogenic pathways identified as key molecular targets of BPs action. Notably, the association between exposure to BPA analogs and incidence of MRD has garnered significant interest. Prior studies, including epidemiological and experimental investigations have suggested that exposure to BPB or BPS has emerged as a significant environmental factors contributing to various forms of MRD in prepubertal, adolescent, and adult testes, including cryptorchidism, TT, PE, and ED [33,34,35,36]. However, the molecular mechanisms underlying this association remain poorly understood in middle-aged males. This study aims to provide new scientific evidence for assessing the safety of BPs and elucidate the molecular pathogenesis through which BPB and BPS impact MRD in middle-age humans, by integrating findings from network toxicology, molecular docking techniques and in vivo research.

We identified three common genes associated with these four diseases via PPI analysis: AKT1, MYC, and TP53. Previous study has suggested that members of the p53 family are implicated in the survival of male germ cells [37]. Furthermore, p53 modulated the transcription of genes governing major defenses against tumor growth by binding to specific response elements in DNA [38]. Low p53 expression is typically observed in seminomas, whereas high p53 expression is more commonly associated with non-seminomas [39]. AKT1 plays a crucial role in downstream signaling influencing sex hormone signal transduction, consistent with our results. The MYC family proteins affect protein translation, cell-cycle progression and stromal cell metabolism [40]. Given that AKT1, MYC, and TP53 are hub genes associated with the four MRD conditions, it is reasonable to hypothesize that patients with any of these diseases may exhibit altered expression of these genes, and such differential expression could drive the development of another disease, consistent with our findings of significant correlations among these diseases.

Several core signaling pathways are closely associated with MRD, including “cancer-related pathways”, “signaling regulatory pathways” and “viral infection and epigenetic pathways”. Cancer-related pathways encompass key mechanisms such as PI3K/Akt, MAPK, p53, Wnt, and cell cycle regulation, which are involved in testicular cell proliferation, apoptosis, and endocrine control. Notably, prostate cancer is closely linked to testosterone imbalance and aberrant androgen receptor (AR) signaling. BPB and BPS can alter hormonal responses, thereby promoting prostate cancer cell proliferation, disrupting AR signaling, and inhibiting the expression of tumor suppressor genes; long-term exposure is associated with increased susceptibility to precancerous lesions and carcinogenesis. Signaling regulatory pathways play critical roles in hormone synthesis and testicular development. Dysfunction in this pathway implies dysregulated testosterone synthesis, representing a potential mechanism underlying hormonal disorders associated with ED and cryptorchidism. Viral infection and epigenetic pathways are involved in sperm quality and reproductive development. Testis is vulnerable to viral infections, which can trigger local inflammation and disrupt barrier functions. MicroRNAs are confirmed to regulate testicular development, spermatogenesis, and hormone production, while cellular senescence pathways contribute to age-related testicular degeneration and declining androgen levels. Bioinformatics analysis revealed that these four MRD conditions exhibited similar gene expression patterns following exposure to BPs.

Our data indicated that a single high-dose gavage administration of BPB or BPS resulted in detectable residues in the testis, which were more difficult to eliminate than those in the serum, a finding consistent with research on BPAF [41,42]. We further investigated their reproductive toxicity and results demonstrated that middle-aged male mice exposed to BPB and BPs exhibited no significant changes in body weight or relative testis weight, consistent with previous studies on BPA [43]. The testis is a major target organ for EDCs, and testicular morphological changes indicate physiological, functional, or even pathological disorders [44]. BPB or BPS altered testicular morphology, the growth rate of reproductive organs, and impaired sperm production (Figure 8A,D,E). Specifically, high-dose exposure (0.6 mg/kg body weight of BPB or BPS) induced histological alterations in the testes, characterized by reduced sperm count in the vas deferens and enlarged seminiferous tubule diameter. This finding is consistent with previous observations in rats treated with BPA, indicating a detrimental impact on male reproductive health [45]. Seminiferous tubules are the basic structural unit of the testis, and abnormalities in tubule can disrupt spermatogenesis and steroid production, leading to reproductive complications.

Infertility rates have exhibited a significant increase in recent years, currently impacting approximately 15% of the global population, with male factors accounting for nearly half of these cases. Endocrine-disrupting chemicals (EDCs) are defined as exogenous substances that interfere with hormone synthesis, secretion, transport, binding, action, or elimination [46]. Numerous studies have raised concerns regarding their detrimental effects on reproductive health, documenting a decline in human sperm concentration over the past four decades [20,47,48]. The hypothalamic-pituitary-gonadal (HPG) axis plays a critical role in regulating male reproductive function, and EDCs that function as anti-androgens or estrogen mimetics may disrupt this regulatory system, thereby impairing spermatogenesis, sperm motility, and morphology [49]. Consequently, EDCs have been implicated in various manifestations of MRD, including cryptorchidism, testicular tumors, premature ejaculation, and erectile dysfunction. For instance, prenatal exposure to EDCs can interfere with androgen signaling pathways, hinder testicular descent, and increase the risk of testicular cancer later in life, likely through sustained hormonal dysregulation and altered cellular homeostasis [50,51,52]. In the prostate gland, EDCs such as BPA have been reported to facilitate carcinogenesis by modulating local androgen and estrogen synthesis, altering gene expression profiles, and inducing chronic low-grade inflammation [53,54]. Additionally, compounds such as diethylstilbestrol (DES) and genistein may compromise erectile tissue function by disrupting smooth muscle contractility and modulating ER-α expression [55]. Bisphenols, including BPA, BPB, and BPS, interfere with steroidogenic pathways and androgen/estrogen signaling. BPA has demonstrated binding affinity for estrogen receptors ER-α and ER-β, AR, thyroid hormone receptor (TR), and peroxisome proliferator-activated receptor gamma (PPAR-γ), underscoring its extensive endocrine-disrupting potential [56,57,58]. Substitutes for BPA, such as BPB and BPS, have also been associated with reproductive effects, including modulation of *COL1A1* and *COL1A2* gene expression, which may contribute to premature ejaculation [59]. Compared to other endocrine disruptions, the effects of bisphenol substitutes appear multifaceted, influencing both steroidogenic enzyme expression and hormone receptor signaling pathways. Nevertheless, the mechanisms underlying bisphenol-associated MRD in middle-aged men remains inadequately elucidated.

Testosterone (T) levels in our study were obviously lower than those reported in other studies [60,61]. This discrepancy may stem from differences in the age of the mice used: sex hormone levels in middle-aged male mice undergo significant changes, with a notable decline in T levels [62,63]. Our results also suggested that a trend toward decreased T and increased E2 levels in middle-aged male mice after exposure to BPB or BPS, which is consistent with other studies [60]. The ratio of E2/T increased significantly, and this ratio is strongly associated with male reproductive behaviors [64]. In this study, exposure to 0.6 mg/kg/day BPB or BPS for 20 days reduced T levels by 40.85% and 57.34%, respectively, indicating that T may be more sensitive to BPS. The biosynthesis of T and E2 starts from cholesterol, which is cleaved by the P450 side-chain cleavage enzyme (encoded by CYP11A1) to form pregnenolone. Pregnenolone then act as a substrate of the enzyme P450C17 (encoded by CYP17A1), yielding dehydroepiandrosterone (DHEA). Through the action of enzymes from the 17β-hydroxysteroid dehydrogenase (17β-HSD) family and the 3β-hydroxysteroid dehydrogenase (3β-HSD) family, DHEA is converted into testosterone. In terms of estrogen synthesis, aromatase (encoded by CYP19A1) catalyzes the aromatization of testosterone into E2 [54]. In our study, low-dose (0.3 mg/kg b.w.) BPB or BPS treatment was sufficient to reduce the genes expression of testicular steroidogenic enzymes (including CYP11A1, CYP17A1, CYP19A1, HSD3B1, and HSD17B3). Meanwhile, reduced steroid synthase expression supports the histological changes in the testis, including shape of the tubule in the testicular cavity, and the decrease in sperm production after BPB or BPS treatment. Furthermore, these targets were selected for molecular docking with BPB and BPS. Our results revealed that strong binding affinities with CYP11A1, CYP17A1, CYP19A1, HSD3B1, and HSD17B3, suggesting that BPB and BPS may directly interact with these key steroidogenic enzymes.

The relationship between exposure to BPs and MRD remains a significant research challenge. In our study, we identified potential targets and molecular mechanisms underlying the induction of MRD by BPB and BPS exposure. Importantly, our results demonstrated that exposure to BPB or BPS exerts deleterious effects on reproductive function in middle-aged male mice. Notably, BPB and BPS exert endocrine-disrupting properties affect testicular morphology, sex hormone levels, and steroidogenic enzyme expression in middle-aged male mice. However, the effects of BPB and BPS on steroidogenic enzymes have been inferred from in vivo expression analyses and molecular docking simulations, and direct experimental validation of their binding interactions is lacking. To address this research gap, subsequent biochemical or biophysical assays—including co-immunoprecipitation (Co-IP), surface plasmon resonance (SPR), and nuclear magnetic resonance (NMR) spectroscopy—should be performed to verify these interactions. This investigation elucidates that exposure to BPB and BPS compromise testicular function and perturbs the steroidogenic pathway in adult murine models, indicating a potential hazard for reproductive dysfunction in middle-aged males. Employing an integrative approach that combines network toxicology, molecular docking, and in vivo experimentation, the study identifies plausible mechanistic associations between BPs and four prevalent MRDs, thereby contributing substantive insights to the disciplines of toxicology and environmental health. While these findings derive from rodent models and computational predictions, necessitating further corroboration through epidemiological and clinical investigations, they underscore the imperative to reevaluate the safety profiles of BPB and BPS. From a public health standpoint, systematic surveillance of the environmental distribution of bisphenol analogs and their potential repercussions on male reproductive health bears considerable societal significance. Collectively, this research furnishes a comprehensive framework elucidating the role of BPB and BPS in MRDs and establishes a foundational basis to inform subsequent inquiries into the reproductive risks posed by bisphenol substitutes.

## 4. Materials and Methods

### 4.1. Animals and Treatment Procedure

Male CD-1 mice were purchased from Beijing Vital River Laboratory Animal Technology Co., Ltd. (Beijing, China) and kept in standard light- (12L:12D cycles) and temperature (25 ± 3 °C)-controlled cages. Mice were given with a standard animal diet and purified water in glass bottles ad libitum. The age of mice at the time of administration was 5-month-old.

For the single gavage, CD-1 mice were randomly divided into fifteen groups (*n* = 6 per group). BPB (CAS NO. 77-40-7; purity ≥ 98%) and BPS (CAS NO. 80-09-1; purity: 99%) were obtained from Shanghai Aladdin Biochemical Technology Co., Ltd. (Shanghai, China), dissolved in corn oil (C116025, Shanghai Aladdin Biochemical Technology Co., Ltd.) and stored in glass vials. Each animal was weighed prior to administration and body weight was used to determine the dose. The design of experimental sampling time is 0, 4, 8, 12, 32 h. Experimental sampling was scheduled at 0, 4, 8, 12, and 32 h post-administration. At each time point, mice were euthanized to collected blood and testes. Corn oil was used as the negative control.

The selected doses were lower than both the no-observed-adverse-effect level (NOAEL, 5 mg/kg/day) and the lowest-observed-adverse-effect level (LOAEL, 50 mg/kg/day) specified by Environmental Protection Agency (EPA) for BPA. No NOAEL and LOAEL have been established for BPB and BPS. Therefore, 0.3 mg/kg/day represents an environmentally relevant and biologically active dose, while 0.6 mg/kg/day provides a moderate escalation to explore potential dose-dependent effects [65]. For the sub-chronic gavage administration, male mice were randomly divided into six groups (*n* = 6 per group): 0 mg/kg body weight (b.w.) (control), 0.3 mg/kg b.w. BPS, 0.6 mg/kg b.w. BPS, 0.3 mg/kg b.w. BPB, 0.6 mg/kg b.w. BPB, and 0.3 mg/kg b.w.17β-estradiol (E2). Corn oil was served as solvent and E2 was used as the positive control. Over 20 consecutive days, the body weight and general condition of each mice were recorded prior to each gavage. On the morning of the 21st day, mice were weighed and then euthanized. Blood samples were collected, and testes were dissected: one testis was fixed in 4% paraformaldehyde, while the other was stored at −80 °C for RT-qPCR analysis. To increase the rigor of the analysis, testis and serum nuclear samples were processed in a double-blind manner until final statistical analysis. All experimental procedures were approved by the Institutional Animal Care and Use Committee of Shenyang Agricultural University (Approval No. 24031201).

### 4.2. Network Toxicological Analysis of BPB and BPS

The SMILE sequences of BPB and BPS were initially retrieved from the PubChem database (https://pubchem.ncbi.nlm.nih.gov/ (accessed on 17 April 2025)). For consistency, IDs of protein and gene were obtained from the UniPort database (https://www.uniprot.org/ (accessed on 17 April 2025)). The keywords “Cryptorchidism”, “Erectile Dysfunction”, “Testicular Tumor”, “Premature Ejaculation”, “Bisphenol B” and “Bisphenol S” were queried in GeneCards database (https://www.genecards.org (accessed on 17 April 2025)), with the species set to *Homo sapiens* (Human). To ensure the obtained genes were highly pertinent to both “Male reproductive dysfunction” and the effects of BPB/BPS (BPs), a filtering threshold based on the median relevance score from GeneCards database was applied. Only genes with relevance scores exceeding the median threshold were retained for analysis. The intersection between MRD targets and BPs targets was identified and visualized using R. studio, and the pollutant-target network was subsequently constructed utilizing Cytoscape software (version 3.9.0).

### 4.3. Construction of Protein–Protein Interaction (PPI) Networks

The PPI network was established utilizing the STRING database (https://cn.string-db.org/ (accessed on 17 April 2025)), incorporating overlapping targets identified from the Venn diagram analysis. The species was specified as *Homo sapiens,* and an interaction confidence score threshold of ≥0.7 was constructed for network assembly. The resulting network was then imported into Cytoscape software (version 3.9.0) for visualization and analysis, with the single notes being deleted.

### 4.4. Bioinformatic Analysis

Gene Ontology (GO) together with Kyoto Encyclopedia of Genes and Genomes (KEGG) analyses were performed utilizing Metascape (https://metascape.org (accessed on 22 April 2025)), incorporating overlapping targets identified from the Venn diagram analysis. GO analysis classified enriched terms into three categories: biological processes (BP), cellular components (CC), and molecular functions (MF), providing insights into the functional roles of potential targets. KEGG enrichment identified MRD-related pathways with a threshold of <0.05, aiming to elucidate and highlight signaling pathways implicated in MRD via core targets. All results were plotted using R. studio (version 4.1.0) for data analysis and visualization.

### 4.5. Molecular Docking

The structures of BPB and BPS were obtained from the PubChem database (https://pubchem.ncbi.nlm.nih.gov/ (accessed on 23 April 2025)). The three-dimensional protein structures of AKT1, MYC, TP53, CYP11A1, CYP17A1 and CYP19A1 were downloaded from the PDB database (https://www.rcsb.org/ (accessed on 23 April 2025)). The three-dimensional structure of HSD3B1 and HSD17B3 were predicated using the AlphaFold3 Protein Structure Database (https://alphafold.ebi.ac.uk/ (accessed on 23 April 2025)). The protein structures were obtained with the species parameter specified as *Homo sapiens* (Human). AutoDock Vina 4.2 was used to predict the binding affinity between the putative targets and BPB/BPS. Root-mean-square deviation (RMSD) was used to calculate and analyze the molecular dynamics trajectories. PyMOL (version 2.5.4.0) was utilized to visualize and analyze interactions and binding modes.

### 4.6. Quantitation of BPS or BPB by UPLC-MS/MS from Single Gavage

Testis and blood samples were extracted by liquid–liquid extraction after preparation [66]. Extraction was repeated three times with ethyl acetate. Subsequently, the mixture was vortexed at 110 rpm for 4 min, and the volume was fixed with methanol. The results were analyzed by Ultra Performance Liquid Chromatography Tandem Mass Spectrometry (UPLC-MS/MS). Samples were analyzed for BPB or BPS using a validated analytical method. BPB and BPS were, respectively, dissolved in methanol to prepare 1 mg/mL stock solution and then diluted to concentrations of 0.005 μg/mL, 0.05 μg/mL, 0.5 μg/mL, 5 μg/mL, and 10 μg/mL for the determination of standard curve.

### 4.7. Histological Analysis

Six mouse testes from each group were collected and fixed in 4% paraformaldehyde for 48 h. As previously described, the paraffin sections (5 μm) were stained with hematoxylin and eosin for histological analysis [67]. Four sections were selected from each group, and six fields in each section were randomly observed. The average diameter and circumference of the seminiferous tubules were measured and calculated using NIS-Elements software (version 4.50; Nikon, Tokyo, Japan) for morphological analysis.

### 4.8. ELISA Hormone Assays

The serum of sub-chronically intragastrically gavaged mice was analyzed. 17β-estradiol (E2) and testosterone (T) were measured using enzyme-linked immunosorbent assay (*ELISA*) kits purchased from Elabscienc Biotechnology Co., Ltd. (Wuhan, China), according to the manufacturer’s instructions. The sensitivity of each ELISA kit was 1.88 pg/mL for E2 and 0.14 ng/mL for T, respectively.

### 4.9. RNA Extraction and Quantitative Real-Time PCR (RT-qPCR)

Total RNA was extracted from testis by Trizol reagent (TaKaRa Bio Inc., Shiga, Japan), and then cDNA was synthesized using Go Scription™ Reverse Transcription System (Shanghai Promega Biological Products Co., Ltd., Shanghai, China). RT-qPCR was performed using a CFX96 Real-Time PCR Detection System (BioRad, Shanghai, China) with TB Green^®^ Premix Ex Taq™ II (Takara, Dalian, China) as the detector. Primer sequences for the genes are provided in Table S1. The PCR cycle parameters were as follows: 95 °C for 5 s and 60 °C for 30 s, repeated for 40 cycles. Template-free PCR reactions were used as negative controls to verify experimental results. Glyceraldehyde-3-phosphate dehydrogenase (*Gapdh*) served as the reference gene, with values from the control group used for normalization. Data were normalized against *Gapdh* and calculated using 2^−ΔΔCT^ method [68].

### 4.10. Statistical Analysis

Each experimental procedure was conducted independently at least three times. Data were analyzed using one-way ANOVA (Tukey’s multiple comparisons test) in GraphPad Prism 10 (GraphPad Software Inc., San Diego, CA, USA). Data were expressed as the mean ± standard error of the mean (SEM). A *p*-value of less than 0.05 was deemed to indicate statistical significance. Significant differences are defined as * *p* < 0.05, ** *p* < 0.01, *** *p* < 0.001, and **** *p* < 0.0001.

## 5. Conclusions

In conclusion, our study sheds light on the potential mechanistic links between BPs and four prevalent MRD conditions, contributing valuable insights to the fields of toxicology and environmental health. By integrating network toxicology, molecular docking techniques and in vivo experiment, these findings provide a comprehensive understanding of how BPB and BPS influence MRD in middle-aged males. The results underscore the necessity of assessing the safety of BPB and BPS, and provide a theoretical framework as well as valuable insights for future research.

## Figures and Tables

**Table 1:** The Lowest Binding Energy Between BPs and Potential Receptors.

BPB		BPS	
Protein Name	Affinity (kcal/mol)	Protein Name	Affinity (kcal/mol)
AKT1	−6.0	AKT1	−4.5
MYC	−5.9	MYC	−5.6
TP53	−7.3	TP53	−7.6

**Table 2:** The Lowest Binding Energy Between BPs and Human Steroidogenic Enzyme.

BPB		BPS	
Protein Name	Affinity (kcal/mol)	Protein Name	Affinity (kcal/mol)
CYP11A1	−6.9	CYP11A1	−2.2
CYP17A1	−6.7	CYP17A1	−7.7
CYP19A1	−6.6	CYP19A1	−5.8
HSD3B1	−5.4	HSD3B1	−4.9
HSD17B3	−5.1	HSD17B3	−5.1

## Data Availability

The authors confirm that the data supporting the findings of this study are available within the article. If someone wants to request the Data from this study, please contact yumingxi2015@syau.edu.cn.

## Supplementary Materials

The following supporting information can be downloaded at https://www.mdpi.com/article/10.3390/ijms26199507/s1.
