# Supplementary material for: Exposure to Bisphenol B and S Increases the Risk of Male Reproductive Dysfunction in Middle Age
Source: Int J Mol Sci. 2025 Sep 28;26(19):9507. doi: 10.3390/ijms26199507 (PMC12525246; doi:10.3390/ijms26199507)
Supplement: Supplementary file 1 [file ijms-26-09507-s001.zip › Table S1 List of RT-qPCR primers used in this paper.pdf]

**Table S1.** List of RT-qPCR primers used in this paper.		
**Gene**	**Forward**	**Reverse**
Gapdh	GTGTTCCTACCCCCAATGTGT	ATTGTCATACCAGGAAATGAGCTT
Cyp11al	TGTGATTTTCAATAAAGCTGATGA	TTCTTGAAGGGCAGCTTGTT
Cyp17al	TTTATGCCTGAGCGCTTCTT	GCAGCAAGGCCATGAAGATA
Cyp19al	CTGTTGTGGGTGACAGAGACA	GCCGTCAATTACGTCATCCT
Hsd3b1	AACAATTTAACAGCCCTCCTAAG	GCACCAACATCTTGATGATCC
Hsd17b3	TCAGCTTCCAAGGCTTTTGT	GGTACTTTGTCATTGGGGTTG
